# Functional Ultrasound (fUS) During Awake Brain Surgery: The Clinical Potential of Intra-Operative Functional and Vascular Brain Mapping

**DOI:** 10.3389/fnins.2019.01384

**Published:** 2020-01-09

**Authors:** Sadaf Soloukey, Arnaud J. P. E. Vincent, Djaina D. Satoer, Frits Mastik, Marion Smits, Clemens M. F. Dirven, Christos Strydis, Johannes G. Bosch, Antonius F. W. van der Steen, Chris I. De Zeeuw, Sebastiaan K. E. Koekkoek, Pieter Kruizinga

**Affiliations:** ^1^Department of Neurosurgery, Erasmus MC, Rotterdam, Netherlands; ^2^Department of Neuroscience, Erasmus MC, Rotterdam, Netherlands; ^3^Department of Biomedical Engineering, Thorax Centre, Erasmus MC, Rotterdam, Netherlands; ^4^Department of Radiology and Nuclear Medicine, Erasmus MC, Rotterdam, Netherlands; ^5^Netherlands Institute for Neuroscience, Royal Dutch Academy for Arts and Sciences, Amsterdam, Netherlands

**Keywords:** brain tumor, functional ultrasound, awake craniotomy, tumor vasculature, neoplasm, imaging

## Abstract

**Background and Purpose:**

Oncological neurosurgery relies heavily on making continuous, intra-operative tumor-brain delineations based on image-guidance. Limitations of currently available imaging techniques call for the development of real-time image-guided resection tools, which allow for reliable functional and anatomical information in an intra-operative setting. Functional ultrasound (fUS), is a new mobile neuro-imaging tool with unprecedented spatiotemporal resolution, which allows for the detection of small changes in blood dynamics that reflect changes in metabolic activity of activated neurons through neurovascular coupling. We have applied fUS during conventional awake brain surgery to determine its clinical potential for both intra-operative functional and vascular brain mapping, with the ultimate aim of achieving maximum safe tumor resection.

**Methods:**

During awake brain surgery, fUS was used to image tumor vasculature and task-evoked brain activation with electrocortical stimulation mapping (ESM) as a gold standard. For functional imaging, patients were presented with motor, language or visual tasks, while the probe was placed over (ESM-defined) functional brain areas. For tumor vascular imaging, tumor tissue (pre-resection) and tumor resection cavity (post-resection) were imaged by moving the hand-held probe along a continuous trajectory over the regions of interest.

**Results:**

A total of 10 patients were included, with predominantly intra-parenchymal frontal and temporal lobe tumors of both low and higher histopathological grades. fUS was able to detect (ESM-defined) functional areas deep inside the brain for a range of functional tasks including language processing. Brain tissue could be imaged at a spatial and temporal resolution of 300 μm and 1.5–2.0 ms respectively, revealing real-time tumor-specific, and healthy vascular characteristics.

**Conclusion:**

The current study presents the potential of applying fUS during awake brain surgery. We illustrate the relevance of fUS for awake brain surgery based on its ability to capture both task-evoked functional cortical responses as well as differences in vascular characteristics between tumor and healthy tissue. As current neurosurgical practice is still pre-dominantly leaning on inherently limited pre-operative imaging techniques for tumor resection-guidance, fUS enters the scene as a promising alternative that is both anatomically and physiologically informative.

## Introduction

Oncological neurosurgery relies heavily on making continuous, intra-operative delineations between tumor and brain tissue. The ultimate surgical aim is reaching maximum-safe tumor resection, in which most of the tumor is removed, while preserving functional brain areas to prevent post-operative neurological and cognitive deficits (Marko et al., 2014; Li et al., 2015). Given the large heterogeneity in tumor presentation and growth, especially in gliomas (Patel et al., 2014), optimal delineation remains a challenging task, even when done through a high-resolution surgical microscope. Therefore *in vivo* real-time, functional brain imaging is essential to advance maximum-safe tumor resection to the next level.

Conventional clinical practice makes use of several pre-operative imaging modalities, including (f)MRI and DTI, which are linked to intra-operative neuro-navigation systems. These imaging modalities allow the surgeon to pre-operatively acquire anatomical and functional data of the tumor and surrounding eloquent areas. However, due to the inevitable brain shift after cranio- and durotomy, pre-operative images only provide a rough estimation of the 3D-locus of the tumor during *in vivo* surgery following craniotomy (Imbault et al., 2017; Fabelo et al., 2018), complicating tumor delineation as the surgery proceeds. In some specialized hospitals, MRI is also available intra-operatively (iMRI) in so called ‘MRI surgical suites.’ Although the use of iMRI can solve initial brain shift-related problems, it does not allow us to readily distinguish surgically manipulated healthy brain tissue, edema, and tumor-infiltrated brain tissue. Furthermore, iMRI disrupts surgical workflow, is time-consuming, and requires a large financial investment.

The widespread introduction of awake craniotomy surgery with ESM has greatly improved the intra-operative identification of eloquent brain areas adjacent to tumors. Intra-operative use of ESM to remove low- (De Witt Hamer et al., 2012) or HGGs (Gerritsen et al., 2019) has been associated with fewer post-operative complications and higher percentages of GTRs. However, ESM also presents with a range of its own inherent limitations, including lack of (sulcus-)depth resolution (Imbault et al., 2017), functional over-estimation due to current leakage (Ritaccio et al., 2018), lack of standardization of stimulation protocols (Pouratian et al., 2004; Ritaccio et al., 2018) and the risk of eliciting epileptic seizures (Su and Ojemann, 2013; Ritaccio et al., 2018).

Other conventional and experimental imaging techniques such as Laser Speckle Contrast Imaging (LSCI) (Klijn et al., 2012), Optical Coherence Tomography (OCT) (Valdés et al., 2016; Almasian et al., 2019) Near-Infrared Spectroscopy (NIRS) (Murata, 2008), HSI (Fabelo et al., 2018), and FGS (Zhang et al., 2018) also facilitate intra-operative functional and/or anatomical imaging, but they also suffer from critical limitations, such as a limited penetration depth (millimeters), field of view or spatial resolution.

These limitations of currently available pre- and intra-operative imaging modalities greatly warrant the need for the development of new real-time image-guided resection tools, which allow for reliable anatomical and functional information in an intra-operative setting. Recently, such an alternative emerged within the field of ultrasound imaging, to which we refer as fUS. fUS relies on HFR ultrasound and subsequent Doppler processing and interregional correlation analyses (Deffieux et al., 2018). As such, it allows for the detection of very small changes in vascular dynamics including changes in CBV, CBF and related processes such as vasodilation, which in turn reflect changes in metabolic activity of activated neurons through neurovascular coupling (Deffieux et al., 2018). Therefore, fUS can capture brain functionality in real-time by detecting small hemodynamic responses with a temporal resolution in the order of milliseconds, which is believed to be much higher than the vascular dynamics involved in neurovascular coupling (Deffieux et al., 2018; Logothetis, 2018). In addition to its high temporal resolution, fUS can reach a spatial resolution of approximately 50 μm (Imbault et al., 2017), rendering the technique in principle valuable for advancing the borders of maximum-safe tumor resection.

So far, fUS has been applied successfully in brains and spinal cords of mice (Tiran et al., 2017; Koekkoek et al., 2018; Macé et al., 2018; Soloukey et al., 2019), rats (Osmanski et al., 2014; Tiran et al., 2017; Song et al., 2019), ferrets (Demené et al., 2016; Bimbard et al., 2018), birds (Rau et al., 2018), swine (Song et al., 2019), primates (Dizeux et al., 2019), and humans (Imbault et al., 2016, 2017; Demene et al., 2017), proving to be a powerful tool for studying the dynamics of endogenous brain signals. By combining its high spatiotemporal resolution with a relatively large field of view, fUS can also capture functional brain connectivity across many different brain regions in real time. In addition, fUS has shown to be a low-cost, contrast-free, and mobile technique (Deffieux et al., 2018). This unique set of features warrants the exploration of the clinical, intra-operative application of fUS.

In this work, we apply fUS to a cohort of patients undergoing awake neurosurgery following craniotomy for the indication of tumor removal near eloquent areas of the brain. We present a range of anatomically informative images acquired intra-operatively, discussing their potential for both clinical applications and functional brain-mapping. More specifically, we present here for the first time whether fUS can be exploited at the clinical level to reveal tumor-specific *vascular* features and at the functional level to delineate healthy brain activity patterns surrounding the tumor during motor and cognitive (language-related) tasks.

## Materials and Methods

### Inclusion of Participants

After obtaining approval from the METC (MEC-2018-037, NL64082.078.17) and a written informed consent accordingly, participants were included in the study. All participants were recruited from the Department of Neurosurgery of Erasmus MC in Rotterdam. Participants were eligible for inclusion when diagnosed with a brain tumor planned for tumor removal using awake craniotomy surgery with ESM. Participants were excluded if they were < 18 years old. Some patients underwent fMRI pre-operatively per conventional clinical protocol.

### Study Procedure

**FIGURE 1:**
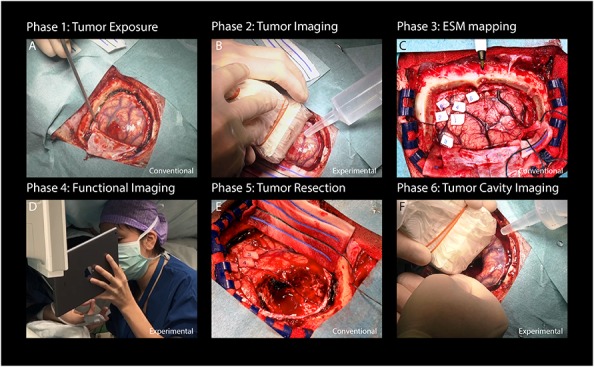
Flowchart providing an overview of the study procedure. **(A)** After conventional craniotomy and durotomy, the tumor was exposed. **(B)** Imaging commenced with a sweeping motion as made by the surgeon across the length of the tumor to capture multiple 2D-images along a continuous trajectory. **(C)** Afterward, conventional ESM was performed to determine eloquent areas of interest surrounding tumor tissue. **(D)** Based on the ESM-defined areas, appropriate functional tasks were presented to the patient. Each functional task consisted of a 60 s task pattern, alternating three functional and rest blocks. Motor and visual tasks were presented on a 12 inch tablet to the patient. Language tasks were only seen by the clinical linguist and communicated verbally to the patient. In this case, a finger tapping motor task is presented to the patient. **(E)** After functional imaging, conventional tumor resection was performed. **(F)** Finally, the resection cavity was filled with saline to ensure adequate acoustic coupling, after which a final acquisition of the resection cavity was made [in a similar fashion as the tumor imaging explained under **(B)**]. Written informed consent was obtained for the identifiable image **(D)**. ESM, electrocortical stimulation mapping.

Functional ultrasound-data acquisition was conducted around three time-points as indicated in Figures 1B,D,F, which in total prolonged the conventional surgical procedure (Figures 1A,C,E) by a maximum of 20 min. After conventional craniotomy and durotomy, the probe (in a sterile cover with ultrasound coupling gel) was placed over the tumor (guided by the neuro-navigation and/or based on visual inspection). A 3D-volume of the tumor was obtained by acquiring 2D-images during a 60 s sweeping motion along a continuous trajectory made by the surgeon. Saline was added frequently to the operating field by the OR nurse to ensure adequate acoustic coupling during imaging.

#### Electrocortical Stimulation Mapping (ESM)

After acquisition of tumor vasculature images, the conventional ESM procedure was performed. Using a bipolar electrode connected to a cortical stimulation unit (Grass Technologies, Astro-Med, Inc.), square-wave pulses were delivered to induce depolarization of relevant cortices. According to standard protocol, the intensity of the working current was increased from 6 to maximum 12 mA (60 Hz, 1 ms), depending on whether a functional effect was evoked. In some cases, no functional effect was found, even at maximum current intensity. Eloquent areas, if found during ESM, were labeled with numbers.

#### Functional Tasks

During fUS-imaging, the probe was partially placed over the (expected location of) the tumor and partially over the surrounding functional areas as identified by ESM, ensuring that both types of tissues were in the field of view. If ESM did not allow us to identify any functional area, the probe was placed over the tumor and surrounding tissue most likely to be functional as extrapolated from the local anatomy.

Based on the expected eloquent area under the probe, patients were presented with appropriate, matching tasks for that area. These tasks were anticipated beforehand as much as possible, based on both anatomy as well as pre-operative fMRI-data if available. Each task consisted of a total of 60 s, with alternating three blocks of tasks (8 s each) and three blocks of resting conditions (10 s each), all preceded by a 6 s baseline. Functional tasks as used in this study ranged from motor tasks (e.g., ‘lip pouting’), to language tasks (e.g., ‘word repetition’) (De Witte et al., 2015) and visual tasks (e.g., ‘8 Hz checkerboard’). Motor and visual tasks were presented on a 12 inch tablet to the patient, including a progress bar for the patient to follow task pattern-timing. Language tasks were only seen by the clinical linguist and communicated verbally to the patient.

Patients were neurolinguistically assessed for the task prior to surgery and informed about the tasks by a clinical linguist, who also presented the tasks to the patients intra-operatively (*DS*). An overview of the details of all the functional tasks as used per patient can be found in Supplementary Table S1.

After functional imaging, tumor resection was commenced. Finally, the tumor resection cavity was filled with saline and a final acquisition was made by the surgeon to capture the remaining tissue vasculature post-resection.

### Image Acquisition

For ultrasound data acquisition, we used an experimental research system (Vantage-256, Verasonics, United States) interfaced with a 5 MHz, 128 element linear array (ATL L7-4, 300 μm pitch) driven with a three cycle burst at 5.2 MHz. Baseband quadrature sampling was applied as implemented by the Verasonics system in order to reduce the overall data-rate. For all scans we acquired continuous angled plane wave acquisition (12–16 angles equally spaced between −12 and 12 degrees) with a PRF ranging from 6 to 8 kHz depending on the imaging depth. The average ensemble size (number of frames used to compute one PDI) ranged between 120 and 140 frames from which the PDIs were computed, providing a live Doppler FR ranging between 3.6 and 4.8 Hz. The PDIs as well as the raw, angle compounded beamformed frames (taken at an FR ranging from 500 to 667 Hz, see Supplementary Table S2) were stored to a fast PCIe SSD hard disk for offline processing purposes. The PDIs were computed using an adaptive SVD clutter filter (Demené et al., 2015). All processing steps, including Fourier domain image reconstruction, compounding, clutter filtering, and storage were done using an inhouse built CPU/GPU code written in C++/CUDA which was interfaced with the standard Verasonics Matlab (MathWorks, Inc.) environment using MEX. In all cases, the probe was hand-held by the surgeon, and placed inside a sterile cover with ultrasound coupling gel. For vasculature imaging, the probe was either moved along a continuous trajectory over the tumor or resection cavity or ‘fanned’ whereby the imaging plane rotates along the array axis. Instead, for functional imaging, the probe was placed over the area of interest and kept stable during the functional measurement. Both vasculature and functional image acquisition sessions consisted of 60 s each.

### Post-processing

Storage of the raw frames allowed for offline optimization of the scan-specific processing parameters that yielded the best vasculature and functional images. In all cases we mapped the images onto a 100 μm grid using zero-padding in the frequency domain. The ensemble size was adaptively set to match one cardiac cycle. To allow for a smooth PD signal over time we applied a 3/4 overlap between consecutive ensembles.

For the functional datasets we applied rigid motion compensation by registering every PDI to the median PDI using the inbuilt matlab function ‘imregtform.m’. To assess the functional signal we bandpass filtered (passband between 0.05 and 0.5 Hz) the PDI stack over time and computed for every pixel the PCC *r* (Macé et al., 2011). The optimal lag (between −2 and 2 s) between the stimulus signal and the recording was chosen empirically based on the overall functional map. Coefficients higher than 0.3 were considered as functionally relevant and displayed on top of the PDI (displayed in gray) using a red/yellow colormap. The mean signal of the functional and remaining pixels was plotted to confirm the validity of the functional signal.

All the post-processing software and the visualization was done in Matlab (MathWorks, Inc.). We used Paraview (Kitware, Inc.), an open-source software tool, for visualizing the 3D vascular scans.

## Results

### Participant Characteristics

Between October 2018 and June 2019, a total of 10 participants (two females, eight males) were included in the study (Table 1). Included participants had a mean age of 42 years (31–56 years), with predominantly intra-parenchymal frontal and temporal lobe gliomas of WHO grades II–IV. No surgical complications occurred, except for two epileptic seizures in two patients (pt#4 and pt#8), one of which was induced by ESM (pt#4).

**TABLE 1:** Clinical characteristics of the six participants in the current study.

**Pt. number**	**Age category**	**Tumor location**	**Tumor type**	**Pre-op fMRI?**	**Others**
1	36–40	Frontal (left)	HGG (GBM)	Y	Re-operation (7 years prior)
2	46–50	Frontal (left)	LGG	N	
3	40–45	Frontal (left)	LGG	N	
4	30–35	Parietal (right)	LGG	Y	Intra-operative seizure
5	56–60	Temporal (left)	HGG (GBM)	Y	
6	40–45	Occipito-parietal (left)	LGG	Y	
7	56–60	Occipito-parietal (left)	HGG (GBM)	N	
8	46–50	Temporal (right)	HGG	N	Pre-operative seizure
9	30–35	Frontal (left)	HGG	N	
10	30–35	Frontal (left)	LGG	N	

*HGG, high grade glioma; GBM, glioblastoma; LGG, low grade glioma.*

### Functional Brain Mapping

A total of 44 functional measurements were performed in 10 patients, ranging from 2 to 5 functional measurements per patient. Of these 44 measurements, 14 involved finger tapping, 6 lip pouting, 18 verbal or silent word or sentence repetition, and 6 visual checkerboard stimulation (see Supplementary Table S1 for an overview of functional tasks used per patient). After post-processing, 9 measurements showed an actual functional signal during motor and language-related tasks. None of the visual functional tasks showed a functional signal. An overview of all measurements is presented in Supplementary Table S2.

#### Motor Tasks

**FIGURE 2:**
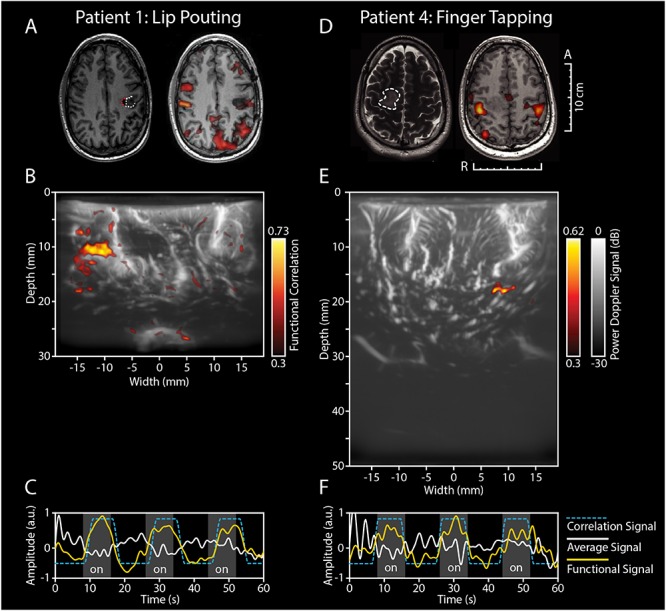
Functional ultrasound results of two functional motor tasks in pt#1 and pt#4. **(A–C)** fUS-results of pt.#1, who presented with a recurrent HGG (GBM) in the left temporal lobe. **(A)** The tumor was located near the left precentral gyrus, as becomes clear from the pre-operative T1-weighted MRI (*left-side*). The white dotted line indicates the tumor borders, with the red dotted line indicating the resection cavity as a result of previous resection. Pre-operative fMRI revealed an association of the tumor with the previous tumor cavity during the functional task of lip pouting, indicating activation of the primary motor cortex of the mouth (*right-side*). **(B)** Intra-operatively, ESM confirmed association with the primary motor cortex. The patient was presented with a 60 s task-video of lip-pouting, consisting of three blocks of tasks (8 s each) and three blocks of resting conditions (10 s each), all preceded by a 6 s baseline. The image depicts the functional correlation map as made during the lip pouting task. A 3.8 cm wide and 3.0 cm deep image reveals functional activity in brain tissue around the tumor cavity evoked by the lip-pouting task. A video of this functional response over time is available as a supplement (Supplementary Video S1). **(C)** As becomes clear from the time traces, the average hemodynamic response in the areas defined as functional in **B**) follows the task pattern (yellow line). In contrast, non-functional areas do not follow this task pattern (white line). Details of this recording session can be found in Supplementary Table S2 (Recording ID 9). **(D–F)** fUS-results of pt.#4, who presented with a LGG in the right parietal lobe. **(D)** The tumor was located in the right parietal lobe, in close proximity to the primary motor cortex, as becomes clear from the pre-operative T2-weighted + GD MRI (*left-side*). The white dotted line indicates the tumor borders. Pre-operative fMRI revealed an association of the overlying motor cortex with the tumor during the functional task of bilateral finger tapping, indicating activation of the primary motor cortex of the hand (*right-side*). **(E)** Intra-operatively, ESM confirmed association with the primary motor cortex of the hand. The patient was presented with a 60 s task-video of finger-tapping, in the same task pattern as described for pt.#1 above. A 3.8 cm wide and 5.0 cm deep image reveals functional activity in deep brain tissue evoked by the lip-pouting task. A video of this functional response over time is available as a supplement (Supplementary Video S2). **(F)** As becomes clear from the time traces, the average hemodynamic response in the areas defined as functional in **(E**) follows the task pattern (yellow line). In contrast, non-functional areas do not follow this task pattern (white line). Details of this recording session can be found in Supplementary Table S2 (Recording ID 26). fUS, functional ultrasound; HGG, high grade glioma; GBM, glioblastoma; ESM, electrocortical stimulation mapping; LGG, low grade glioma; GD, gadolinium.

Figure 2 depicts the fUS-image as acquired from pt.#1 and pt#4, who both performed a motor task intra-operatively. Pt.#1 presented with a recurrent left-sided GBM 7 years after primary surgery. In line with the location of the original and recurrent tumor as well as the images of the pre-operative fMRI, intra-operative ESM confirmed that the primary motor cortex of the mouth was in close proximity to the previous tumor cavity (Figure 2A). After placement of the probe over the relevant ESM-marker, the patient was asked to perform a 60 s lip pouting functional task. The fUS-image allowed for a field of view of 3.8 cm wide and 3.0 cm deep (Figure 2B), in which functional signals related to the task were observed in close proximity to the resection cavity (see also Supplementary Video S1).

Pt.#4 presented with a LGG in the right parietal lobe, also in close proximity to the primary motor cortex, which in pre-operative fMRI was confirmed with a bilateral finger tapping task (Figure 2D). Intra-operatively, ESM again confirmed this association. After placement of the probe over the relevant ESM-marker, the patient was asked to perform a 60 s finger tapping task. The fUS-images allowed for a field of view of 3.8 cm wide and 5.0 cm deep (Figure 2E), in which functional signals related to the task were observed in an area approximately 2.0 cm in depth (see also Supplementary Video S2).

#### Language Tasks

**FIGURE 3:**
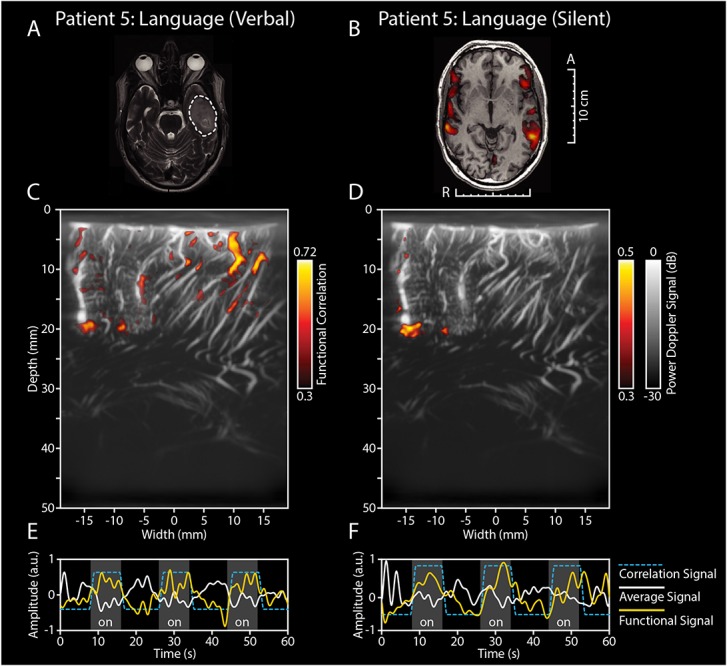
Functional ultrasound results of two functional language tasks in pt#5. fUS-results of two language tasks in pt.#5, who presented with a GBM in the left temporal lobe. **(A)** The tumor was located in the left temporal lobe, as becomes clear from the pre-operative T2-weighted + GD MRI. The white dotted line indicates the tumor borders. **(B)** Pre-operative fMRI data showed an association of the tumor with eloquent areas during a conventional verbal fluency fMRI-task. Intra-operatively, ESM also identified several language-related deficits upon stimulation, including phonemic paraphasia. **(C)** After placement of the probe over the relevant markers, the patient was presented with a word repetition task, which the patient was asked to perform both verbally (by repeating the words out loud) as well as silently (by covertly repeating the words without speaking out loud). In **(C)** the functional correlation map as made during the verbal word repetition task is depicted. The fUS-image allowed for a field of view of 3.8 cm wide and 5.0 cm deep with several functional areas found across the cortex in view. Details of this recording session can be found in Supplementary Table S2 (Recording ID 36). **(D)** The functional correlation map as made during the silent word repetition task in the exact same field of view as discussed in **(C)**. In comparison to the verbal word repetition task, this activation map shows less functional areas found within the field of view. **(E,F)** As becomes clear from the time traces, the average hemodynamic response in the areas defined as functional in **(C,D)** follows the task pattern (yellow line). In contrast, non-functional areas do not follow this task pattern (white line) (see also Supplementary Video S3). Details of this recording session can be found in Supplementary Table S2 (Recording ID 37). fUS, functional ultrasound; GBM, glioblastoma; ESM, electrocortical stimulation mapping; GD, gadolinium.

Figure 3 depicts the functional response to a language-related task in pt.#5, who presented with a GBM in the left temporal lobe (Figure 3A). Initial pre-operative fMRI data showed an association of the tumor with eloquent areas during a conventional verbal fluency fMRI-task (Figure 3B). Intra-operatively, ESM also identified several language-related deficits upon stimulation, including phonemic paraphasia. After placement of the probe over the relevant markers, the patient was presented with a word repetition task. The patient was asked to perform this task both verbally (by repeating the words out loud) as well as silently (by covertly repeating the words without speaking out loud). This combination of tasks allowed for the interrogation of different parts within the language-related functional brain areas. The fUS-image allowed for a field of view of 3.8 cm wide and 5.0 cm deep in both versions of the functional task (Figures 3C,D). In both cases, functional signals were found, with the location and extent of activation visually differing between the verbal and silent word repetition (see also Supplementary Video S3).

### Vascular Mapping

Across the 10 patients, a cumulative total of 30 measurements were made of the pre-resection tumor-vasculature, and a total of 16 measurements of the post-resection cavity. An overview of all measurements is presented in Supplementary Table S2.

#### Maximum Projection Images

**FIGURE 4:**
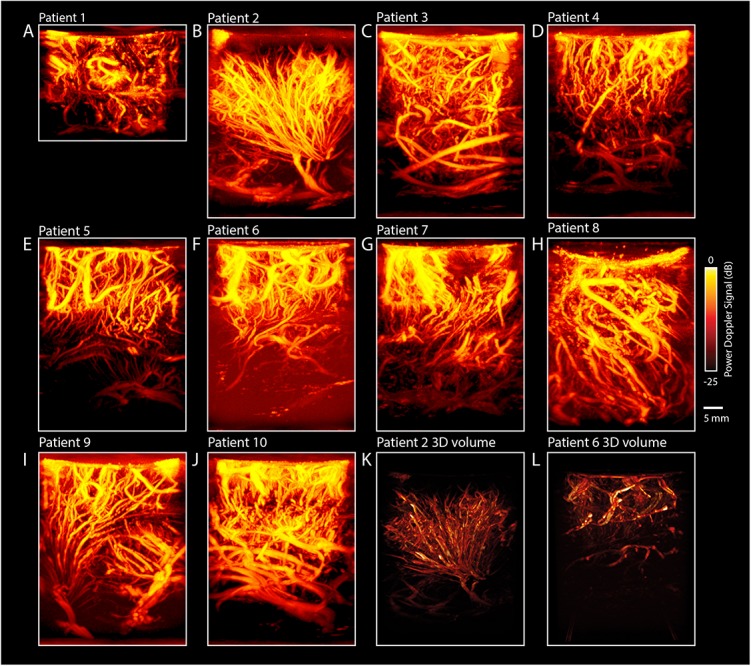
Maximum Projections of all patients (*n* = 10). **(A–J)** In addition to the regular 2D-images, we also made maximum projection images, showing an overview of the maximum signal per pixel during the imaging session of 60 s. As such, a single image with more depth-information can be created. For each patient, the most vascular-dense pre-resection image is highlighted, revealing a rich diversity in vascular patterns across patients. **(K)** 3D-overview of the pre-resection tumor vasculature images as acquired intra-operatively for pt.#2. Pre-operative MRI showed a suspected LGG tumor in the left frontal lobe. The probe was moved over the tumor along a continuous trajectory. During linear 2D-acquisition, an arborization structure was observed of the vessels within the tumor. The arborous vascular structure seems to originate from a single point, dubbed the *vessel of origin*. Multiple 2D PDIs acquired during the 60 s measurement session, showed the arborous structure. The PDIs were stacked offline in a 3D-volume, which confirms the vessel of origin, as depicted here. See also Supplementary Video S4 for the 3D reconstruction. **(L)** Overview of the pre-resection tumor vasculature images as acquired intra-operatively for pt.#6. Pre-operative MRI showed a suspected LGG in the left occipito-parietal region. The probe was moved over the tumor along a continuous trajectory. During linear 2D-acquisition, a well-defined vascular structure was observed, delineating the tumor from the rest of the tissue. Multiple 2D PDIs acquired during the 60 s measurement session, were stacked offline in a 3D-volume, confirming the well-defined vascular area. See also Supplementary Video S5 for the 3D reconstruction. These last two examples highlight the potential of vascular mapping for brain-tumor delineations. fUS, functional ultrasound; LGG, low grade glioma; PDI, power doppler image.

In addition to the regular 2D-images, we also made maximum projection images, showing an overview of the maximum signal per pixel during the imaging session of 60 s. As such, a single image with more depth-information can be created. For each patient, the most vascular-dense pre-resection image is highlighted in Figures 4A–J. As becomes clear from the images, there is a rich variety in tissue vascularization patterns across our patients. In two patients in particular, we saw some interesting tumor vasculature (see Figures 4K,L for reconstructions of 3D-volumes). Tumor-vasculature imaging of the LGG tumor in the left frontal lobe of pt.#2 (Figure 4K) showed an arborous vascular structure, which in 2D-images seemed to originate from a single point, deemed the *vessel of origin*. Offline 3D-reconstruction of the 2D-PDIs confirmed the possible existence of a single vessel of origin in this patient. In pt#6, presenting with a LGG in the occipito-parietal region, tumor vasculature could also be identified. Although not visible to the surgeon on the superficial cortex, the fUS-image pre-resection identified a well-circumscribed oval-shaped region, where the tumor was to be expected (Figure 4L). After using multiple 2D PDIs of the imaging session for reconstruction of a 3D-volume, we observed an oval, well-defined nature of the tumor. These examples highlight the potential of vasculature mapping for brain-tumor delineations. See also Supplementary Videos S4, S5 for the 3D reconstruction and Supplementary Figure S1 for an overview of similar 3D-reconstructions of all patients.

#### Vascular Details

**FIGURE 5:**
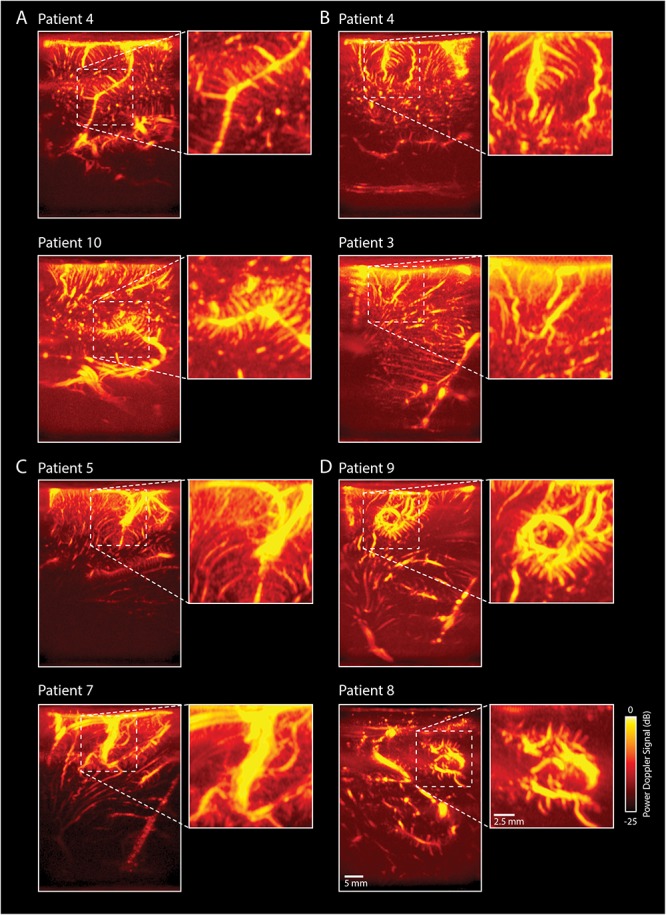
Overview of rich vascular characteristics found in our pre-resection datasets (*n* = 10). **(A)** All patients presented with feather-like vessels, vascular structures consisting of a single, large vessel hosting multiple orthogonal sprouting vessels. These structures were found both in the superficial cortex layers, as well as deeper, and resemble intra-cortical arteries reported previously in literature (Duvernoy et al., 1981). **(B)** Tortuous vessels were also observed. Although the tortuous vessels are known to be potential signs of fast-growing, pathological tumor vasculature, the coiled vessels seem to resemble physiological vascular patterns known as ‘recurrent arteries,’ as also depicted in the SEM-images presented in Duvernoy et al. (1981). These torturous vessels are also visible in **(C)** of this figure (*bottom picture*) and Figure 6 (*patient 4*). **(C)** In those patients where the superficial cortical vessels were exposed, we were also able to capture these larger vessels following, e.g., the sulcus and gyrus patterns of the brain, allowing for lobular distinction. **(D)** Several circular vascular structures were observed, the origins of which remain unknown. The scale bar in the bottom right corner is applicable to all subpanels. SEM, scanning electron microscope.

In addition to the tumor-specific vascular characteristics described above, the pre-resection image acquisition also entailed a rich variety of other high-resolution vascular details in pre-dominantly healthy tissue, some of which are depicted in Figure 5. First, all patients presented with what we dubbed ‘feather vessels’ (Figure 5A), vascular structures consisting of a single, large vessel hosting multiple orthogonal sprouting vessels. These structures were found both in the superficial cortex layers, and more in the depth, resembling intra-cortical arteries reported previously (Duvernoy et al., 1981). In addition, tortuous vessels were also observed (Figure 5B). Although vessel tortuosity is a potential sign of fast-growing, pathological tumor vasculature, the coiled vessels were also observed in healthy tissue, resembling physiological vascular patterns known as ‘recurrent arteries’ (Duvernoy et al., 1981). In those patients where the superficial cortical vessels were exposed, we were often able to follow these larger cortical vessels along the sulci and gyri, some examples of which are depicted in Figure 5C. Lastly, several circular vascular structures were observed (Figure 5D), the origins of which remain unknown. The zoomed-in images give examples of the high resolution (300 μm) and level of detail achieved during image acquisition.

#### Resection-Cavity

**FIGURE 6:**
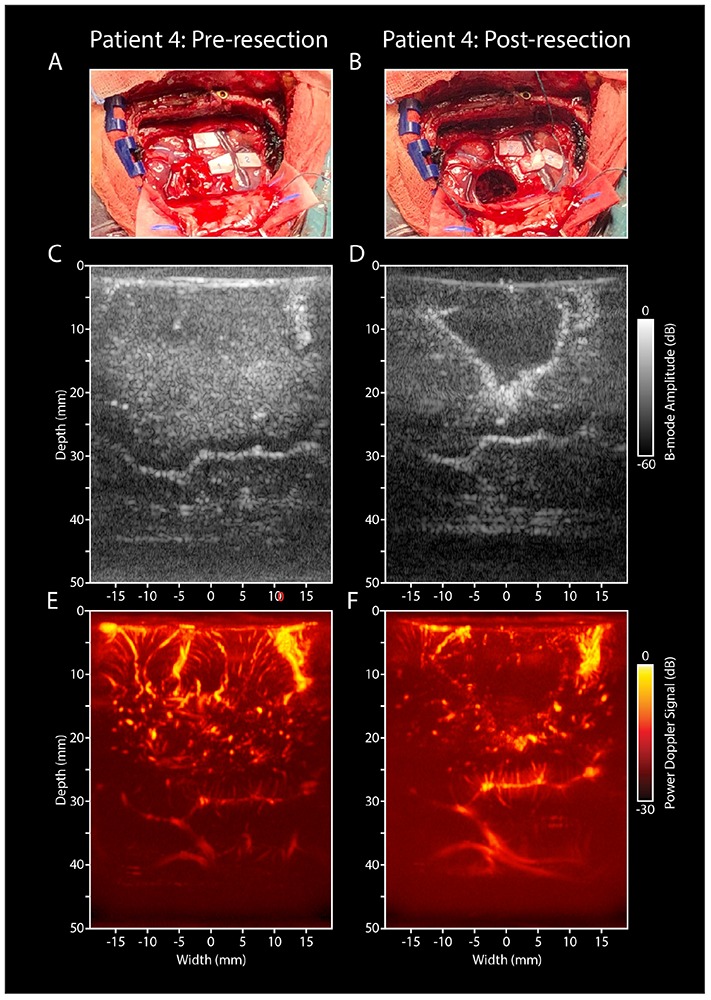
Pre- and post-resection tumor and resection cavity imaging in pt.#4. **(A)** Intra-operative image of a LGG in the right parietal lobe in pt#4, who presented with several eloquent areas in the proximity of the tumor (see ESM-markers). **(B)** Intra-operative image of the post-resection cavity after tumor removal. **(C)** Pre-resection B-mode image of the tumor and surrounding structures. **(D)** Post-resection B-mode image, showing the hypodense resection cavity created after tumor removal. **(E)** PDI of the pre-resection field of view covering the tumor and surrounding healthy tissue. **(F)** PDI of the post-resection field of view depicting the resection cavity, filled with saline. Noticeable are the similar vascular structures depicted in the depth (3–4 cm) of both images **(E,F)**, indicating a similar field of view pre- and post-resection. LGG, low grade glioma; PDI, power doppler image.

Figure 6 depicts an example of a matched pre- and post-resection image of tumor and surrounding healthy tissue in pt.#4. This figure demonstrates the potential of using vasculature characteristics as a guide for tumor-brain delineations intra-operatively, identifying the tumor’s borders based on its vascular characterization.

## Discussion

Inherent limitations of currently available neuro-imaging techniques warrant the development of new real-time image-guided resection tools, which allow for reliable functional and anatomical information in a neurosurgical setting. The present study demonstrates the clinical potential of fUS as a new image-guided resection tool during awake craniotomy surgery. fUS is able to detect the functional areas that were found using ESM (the current gold standard) across a range of functional tasks. As demonstrated by the vascular patterns mapped with submillimeter resolution, fUS also provides a potential new means for tumor delineation based on vasculature characteristics of tumor and healthy tissue.

The work presented here is the second study to demonstrate the power of fUS for neurosurgery as a real-time technique to asses local brain functionality. With respect to the study by Imbault et al. (2016, 2017) we demonstrate the ability of fUS to capture not only motor activation (Figure 2) but also more complex language-related activation (Figure 3). In one of our patients, this was demonstrated by performing a double word repetition language-task, both verbally and silently, which revealed two different activation patterns within the same field of view (Figure 3). Although the exact underlying mechanism remains speculative at this point, the above-mentioned approach using fUS does allow for, e.g., interrogation of the motor-specific and word production component in language. The ability to interrogate the system requires the design of appropriate sets of functional tasks, which became especially apparent in pt.#2 and pt#7 where a verbal language-related task (sentence and word repetition respectively), showed an activation map where language and motor functional response could not be distinguished (Supplementary Figure S2).

In those cases where we did not find functional signals (*n* = 35), the use of an inappropriate functional task could also be a possible explanation. However, problems either due to shifted brain functionalities in space due to oncogenesis, too much in- and out-of-plane motion of the hand-held probe or placement of the probe over an incorrect or non-functional area, form alternative explanations. The latter two explanations are especially plausible, as the majority of the measurements where we found a functional signal (6 out of 9), were made in patients who *did* show ESM-related functional deficits intra-operatively. Furthermore, almost all (8 out of 9) measurements presenting with functional signal showed below average displacement in the x-axis (<0.22 mm), indicating a relatively low level of motion during the measurement (see Supplementary Table S2). Finally, some functional signals presented with unexpected correlation signal timings, starting *before* the actual task pattern (see example of pt#7 in Supplementary Figure S2). Finally, some functional signals presented with unexpected correlation signal timings, starting *before* (see example of pt#7 in Supplementary Figure S2) or *after* the actual the task. As explained in the methods, we aimed to compensate for these differences in timing by allowing a −2 to 2 s delay between the stimulus signal and the recording. Reasons for these lags may include: (1) slow hemodynamic response function, (2) inaccurate timing between start of the acquisition and presented functional task, and (3) delayed or anticipatory response of the patient to the presented task. Determining the potential bias that for example anticipatory effects might have during functional imaging, and how to design proper functional paradigms to exclude them, will be part of our future studies.

The ability of fUS to capture complex functional processes in the brain opens up possibilities not only for functional neurosurgery, but also for unraveling brain function in general. Currently, fUS in humans is still restricted to those circumstances where the brain is no longer covered by skull [craniotomies, fontanels in babies (Demene et al., 2017)]. However, future developments in the field of transcranial-fUS overcoming signal blocking by bone (Errico et al., 2016; Tiran et al., 2017) could open up possibilities for functional studies in humans in a clinical-diagnostic, translational and fundamental setting.

The current study is the first to highlight the potential of real-time, high-resolution imaging of *vasculature* as a means of anatomical delineation. Within clinical oncology, it is widely accepted that tumor angiogenesis and as such tumor vasculature, is differently developed than that of normal tissue (Gerstner et al., 2008; Forster et al., 2017). In fact, numerous therapies such as vascular targeting techniques (Pilat et al., 2004), as well as histopathological malignancy gradings (Hansen et al., 2002; Jain et al., 2007), are based on these tumor-vasculature differences. Using fUS, we have been able to identify tumor-specific vasculature with up to 300 μm resolution in both low and high-grade tumors (finer resolution is achievable at the cost of imaging depth). In one LGG in particular, we were able to image an arborization structure with originating from one *vessel of origin* (Figure 4K). Although our current results are still too preliminary to draw general conclusions against the backdrop of inherent glioma heterogeneity, they do provide ideas for potential fUS applications. For those tumors with vessels of origin, for example, fUS-guided targeted tumor therapies would be an interesting approach. What is more, using our fUS-data in real-time during tumor removal could possibly allow for vascularity-guided tumor resection, in parallel to the treatment of meningiomas with so called ‘pedicles,’ where early access to the vascular origin facilitates safe and effective tumor resection (Dowd et al., 2008; Watts et al., 2014; Hilmani et al., 2016). It would be worthwhile to focus future efforts on imaging heterogeneous groups of brain tumors and comparing and contrasting vascular structure across histopathological gradings, not only in awake but also in anesthetized patients.

Additionally, vascular mapping in our dataset of 10 patients revealed a rich variety of (healthy) vascular detail, including intra-cortical arterial vessels. The resolution with which these vascular images are now made in a real-time, intraoperative setting, is unprecedented. The ability to capture high-resolution vasculature intra-operatively could also have anatomical delineation purposes, not just for tumor-brain delineation, but also delineation of healthy tissue structures, as becomes clear in an incidental imaging session of the thalamus (Supplementary Figure S3). This in turn opens up potential applications in, e.g., neurovascular or functional neurosurgery.

Compared to conventional intra-operative mapping using ESM, fUS provides higher spatiotemporal resolution and higher depth-penetration. In our implementation, temporal resolution can be as high as 1.5 ms, while maintaining real-time display for the surgeon (up to 4.8 Hz) and continuous raw frame storage (up to 667 Hz). Compared to conventional pre- and intra-operative imaging techniques such as (f)MRI, fUS proves to have higher temporal resolution using a more mobile system. Surprisingly, several of our functional imaging sessions showed activation maps also involving concentrated areas at deeper levels of the brain, which would otherwise not have been interrogated intra-operatively with ESM. In addition, the lack of need for electrical stimulation eliminates the risk of intra-operative seizure elicitation. It is also imaginable that several tasks can be performed at once before resecting the tumor, which could save time. Lastly, the movability and cost-effectiveness of fUS make it a highly clinically accessible technique.

Nevertheless, the technique will need to be further validated and improved to reach clinical maturity. For fUS as used in its current form, real-time, automated image classification will be an important next step, which would allow for, e.g., automated brain-tumor delineation. In addition, hand-held fUS-imaging is inherently prone to problems such as motion, both in- and out-of-plane, which can either lead to (1) the inability to capture functional response, or (2) capturing of artifactual ‘functional’ signal. Using linear stages or other probe-mounting options in the OR-setting would be a solution. Future work will also need to focus on the tracking and integration of the probe in currently available neuro-navigation software. Not only will this facilitate the validation of fUS with (f)MRI images made pre-operatively, but it will also allow for better cross-patient comparisons. Comparison of fUS with fMRI in particular is interesting, as both techniques rely on different aspects within the neurovascular coupling mechanism. Where fMRI relies on the BOLD-signal, a measure of blood-oxygenation levels influenced by changes in blood volume and flow as well as the rate of oxygen consumption (Logothetis, 2018) to determine neuronal activity, fUS relies on changes in vascular dynamics as measured by Doppler (Deffieux et al., 2018). How these two measurements of the same phenomenon relate, remains to be elucidated. What is more, it would be worthwhile to critically revisit in future studies our perhaps simplistic assumption of an almost one-to-one correlation between the functional task pattern and the changes in blood dynamics as measured by fUS.

In addition, replacing the currently used linear array with a 3D-probe will allow for intra-operative fUS-imaging of 3D volumes. Both in terms of functional as well as vascular anatomical information, this could constitute a huge improvement. Furthermore, with a 3D-vascular map, vascular-based calibration instead of the usual bone-based calibration for neuro-navigation, could also potentially solve the post-craniotomy brain shift problem. Most importantly, future work will also have to center around the actual patient outcomes in terms of tumor resection, post-operative neurological deficits, and survival when performing fUS-guided resections.

The current study demonstrates that fUS has the potential to be a highly flexible technique for providing vascular as well as functional information in an intra-operative setting with high spatiotemporal resolution. As current neurosurgical practice is still relying on inherently limited imaging techniques for tumor resection-guidance, fUS enters the scene with great clinical potential.

## Data Availability Statement

The raw data supporting the conclusions of this article will be made available by the authors upon reasonable request.

## Ethics Statement

The studies involving human participants were reviewed and approved by the Medical Ethics Review Committee (METC) of the Erasmus Medical Center in Rotterdam, Netherlands (MEC 2018-037). The patients/participants provided their written informed consent to participate in this study.

## Author Contributions

SS, AV, SK, and PK were involved in the study design. SS, AV, DS, and PK were involved in the determination of appropriate intra-operative functional tasks. DS was involved in the intra-operative guidance of the patients while presenting the functional tasks. FM, SS, MS, SK, and PK were involved in the data-processing and -analysis of fMRI and fUS-data. AV included all patients and performed all surgeries and scans. SS, SK, and PK were involved in drafting the manuscript, with the critical input of AV, DS, FM, MS, CMD, CS, JB, AS, and CID.

## Conflict of Interest

The authors declare that the research was conducted in the absence of any commercial or financial relationships that could be construed as a potential conflict of interest.

## Supplementary Material

FIGURE S1 Overview of 3D-volume stacks of patient 1–10. **(A–J)** Multiple 2D PDIs (ranging from *n* = 80–297) acquired during 60 s measurement sessions for each individual patient were stacked offline in a 3D-volume. PDI, power doppler image.

FIGURE S2 Functional ultrasound results of two unusual functional responses to tasks in pt#2 and pt#7 (language). **(A–C)** Functional results of a language task (sentence repetition) in a patient with a LGG in the left frontal lobe. **(A)** Pre-operative MRI showing the extent of the LGG in the left frontal lobe. The white dotted line indicates the tumor borders. **(B)** Intra-operatively, ESM identified both language-related functional areas (anomia), as well as motor related functional areas (primary motor cortex of the mouth) in close proximity to each other. When presented with a verbal functional language task (sentence repetition), multiple functional areas could be defined within the field of view. The lack of a silent language task as a counter-part in this particular patient, complicates the ability to identify between the functional areas in response to the language task vs. the motor activation of the mouth. This in contrast to the functional measurements explicated in Figure 3 with pt.#5. **(C)** As becomes clear from the time traces, the average hemodynamic response in the areas defined as functional in **(B)** follows the task pattern (yellow line). In contrast, non-functional areas do not follow this task pattern (white line). Details of this recording session can be found in Supplementary Table S2 (Recording ID 13). **(D–F)** Functional results of a language task (word repetition, verbal) in a patient with a HGG (GBM) in the occipito-parietal region. **(D)** Pre-operative T2-weighted MRI showing the GBM in the left hemisphere. The white dotted line indicates the tumor borders. **(E)** Intra-operative ESM did not identify any clear functional areas. Based on the anatomical location, the probe was placed partially over a tumor area and partially over a potential functional area related to language. The patient was presented with a verbal functional language task (word repetition), which resulted in an activation map with strong response in the upper left corner of the field of view. Again, the lack of a silent language task as a counter-part in this patient, complicates the ability to identify between the functional areas in response to the language task vs. the motor activation of the mouth. This in contrast to the functional measurements explicated in Figure 3 with pt.#5. **(F)** The time-traces display an unexpected average hemodynamic response in the areas defined as functional in **(E)**, starting *before* the actual task pattern (yellow line). In contrast, non-functional areas do not follow this task pattern (white line). Details of this recording session can be found in Supplementary Table S2 (Recording ID 59). HGG, high grade glioma; GBM, glioblastoma; ESM, electrocortical stimulation mapping; LGG, low grade glioma.

FIGURE S3 Vascular imaging of the thalamus in pt.#10. Incidental imaging session of the thalamus in pt.#10, who presented with a LGG in the left frontal lobe. **(A)** Conventional B-mode image in a sagittal plane, showing (from top to bottom) the corpus callosum, the ventricle, and the thalamus. **(B)** The PDI of the same field of view as described under **(A)**, reveals a rich vascular pattern in the thalamus, allowing for a clear delineation of the nucleus from the surrounding brain tissue. This would open up possibilities for monitoring of vascularization of critical structures such as the thalamus during neurosurgical procedures. **(C)** An overlay of B-mode and PDI, reveals the vascular pattern in relation to the tissue as would be displayed in conventional echography. LGG, low grade glioma; PDI, power doppler image.

TABLE S1 Overview of functional tasks as used in the current study.

TABLE S2 Overview of all recordings (*n* = 90) as performed in the context of the current study.

VIDEO S1 Functional response of pt.#1 during the ‘lip pouting’ functional motor task.

VIDEO S2 Functional response of pt.#4 during the ‘finger tapping’ functional motor task.

VIDEO S3 Functional response of pt.#5 during the ‘word repetition’ functional language task (verbal and silent).

VIDEO S4 3D vascular tumor reconstruction of a LGG in the left frontal lobe of pt.#2.

VIDEO S5 3D vascular tumor reconstruction of a LGG in the occipito-parietal region of pt.#6.

## Abbreviations

BOLD: blood oxygenation level dependent
CBF: cerebral blood flow
CBV: cerebral blood volume
DTI: diffusion tensor imaging
ESM: electrocortical stimulation mapping
FGS: fluorescence guided surgery
fMRI: functional magnetic resonance imaging
FR: frame rate
fUS: functional ultrasound
GBM: glioblastoma
GTRs: gross total resections
HFR: high-frame-rate
HGG: high grade glioma
HIS: hyperspectral imaging
iMRI: intra-operative magnetic resonance imaging
LGG: low grade glioma
LSI: laser speckle imaging
METC: Medical Ethics Review Committee
MRI: magnetic resonance imaging
PCC: Pearson correlation coefficient
PDIs: power doppler images
PRF: pulse repetition frequency
PT: patient
SEM: scanning electron microscope
SVD: singular value decomposition.
