# Supplementary figures and images for: Functional Ultrasound (fUS) During Awake Brain Surgery: The Clinical Potential of Intra-Operative Functional and Vascular Brain Mapping
Source: Front Neurosci. 2020 Jan 9;13:1384. doi: 10.3389/fnins.2019.01384 (PMC6962116; doi:10.3389/fnins.2019.01384)

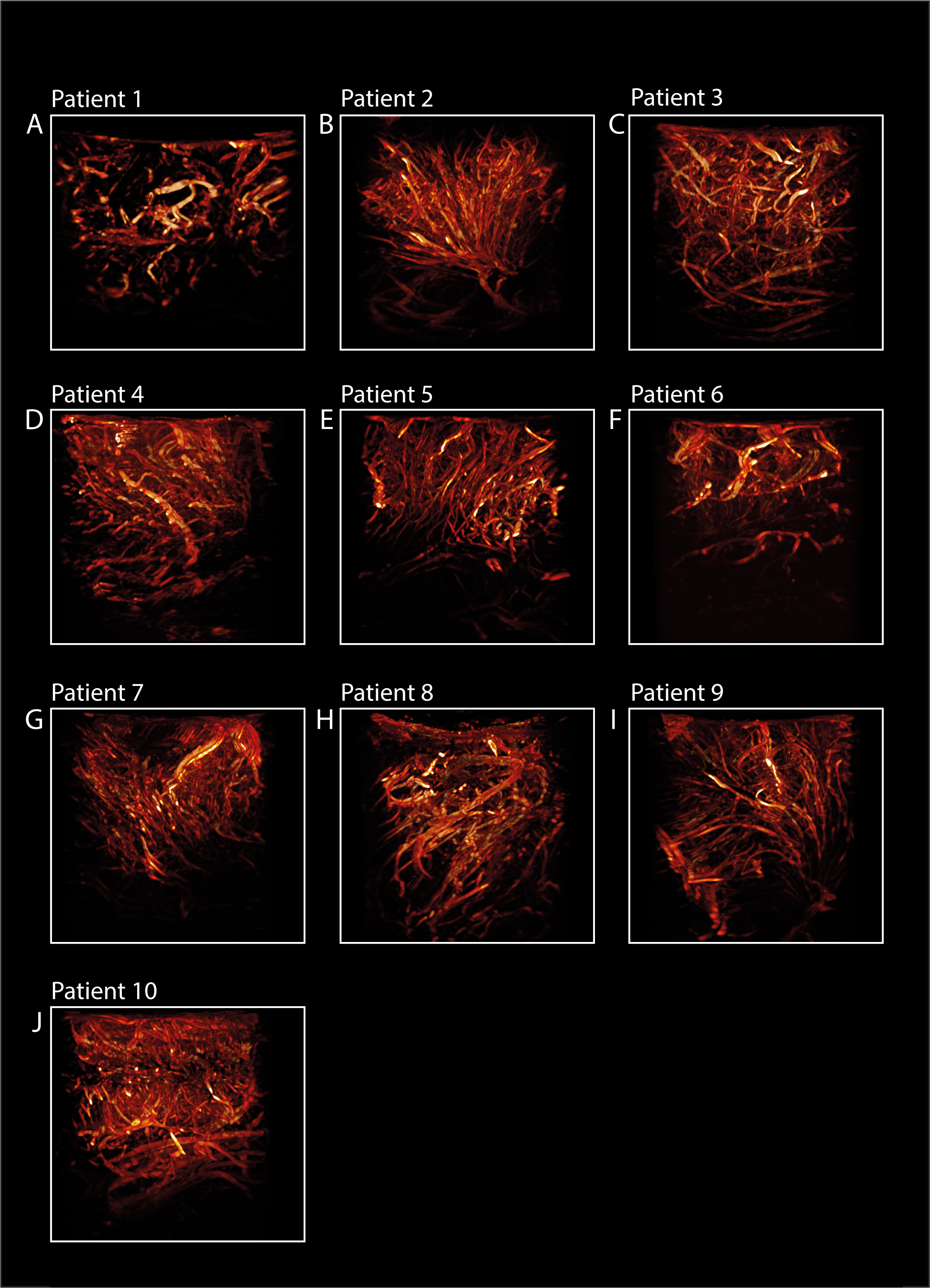

Supplement: FIGURE S1 — Overview of 3D-volume stacks of patient 1–10. (A–J) Multiple 2D PDIs (ranging from n = 80–297) acquired during 60 s measurement sessions for each individual patient were stacked offline in a 3D-volume. PDI, power doppler image. [file Image_1.TIF]

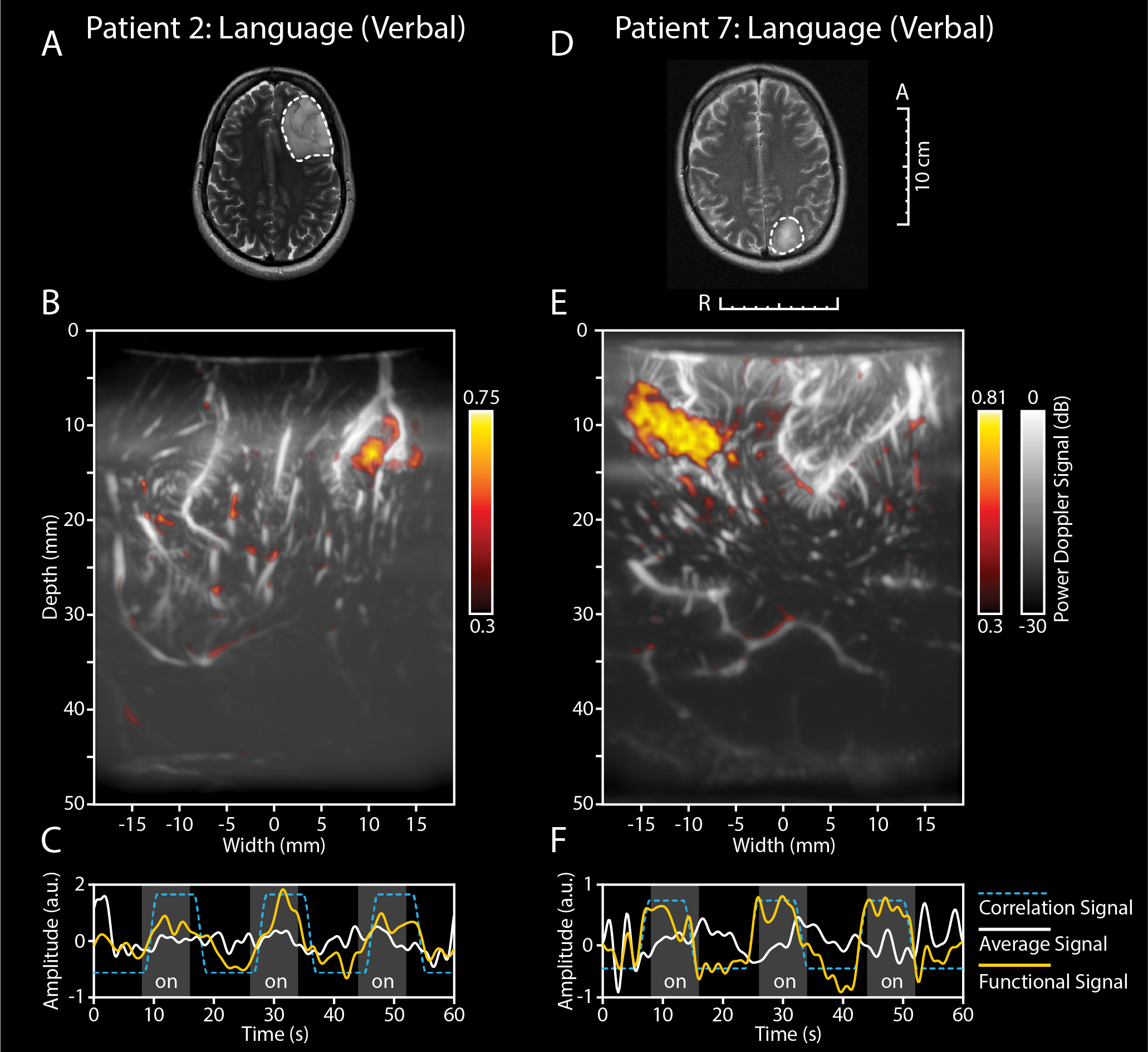

Supplement: FIGURE S2 — Functional ultrasound results of two unusual functional responses to tasks in pt#2 and pt#7 (language). (A–C) Functional results of a language task (sentence repetition) in a patient with a LGG in the left frontal lobe. (A) Pre-operative MRI showing the extent of the LGG in the left frontal lobe. The white dotted line indicates the tumor borders. (B) Intra-operatively, ESM identified both language-related functional areas (anomia), as well as motor related functional areas (primary motor cortex of the mouth) in close proximity to each other. When presented with a verbal functional language task (sentence repetition), multiple functional areas could be defined within the field of view. The lack of a silent language task as a counter-part in this particular patient, complicates the ability to identify between the functional areas in response to the language task vs. the motor activation of the mouth. This in contrast to the functional measurements explicated in Figure 3 with pt.#5. (C) As becomes clear from the time traces, the average hemodynamic response in the areas defined as functional in (B) follows the task pattern (yellow line). In contrast, non-functional areas do not follow this task pattern (white line). Details of this recording session can be found in Supplementary Table S2 (Recording ID 13). (D–F) Functional results of a language task (word repetition, verbal) in a patient with a HGG (GBM) in the occipito-parietal region. (D) Pre-operative T2-weighted MRI showing the GBM in the left hemisphere. The white dotted line indicates the tumor borders. (E) Intra-operative ESM did not identify any clear functional areas. Based on the anatomical location, the probe was placed partially over a tumor area and partially over a potential functional area related to language. The patient was presented with a verbal functional language task (word repetition), which resulted in an activation map with strong response in the upper left corner of the field of view. Again, th [file Image_2.TIF]

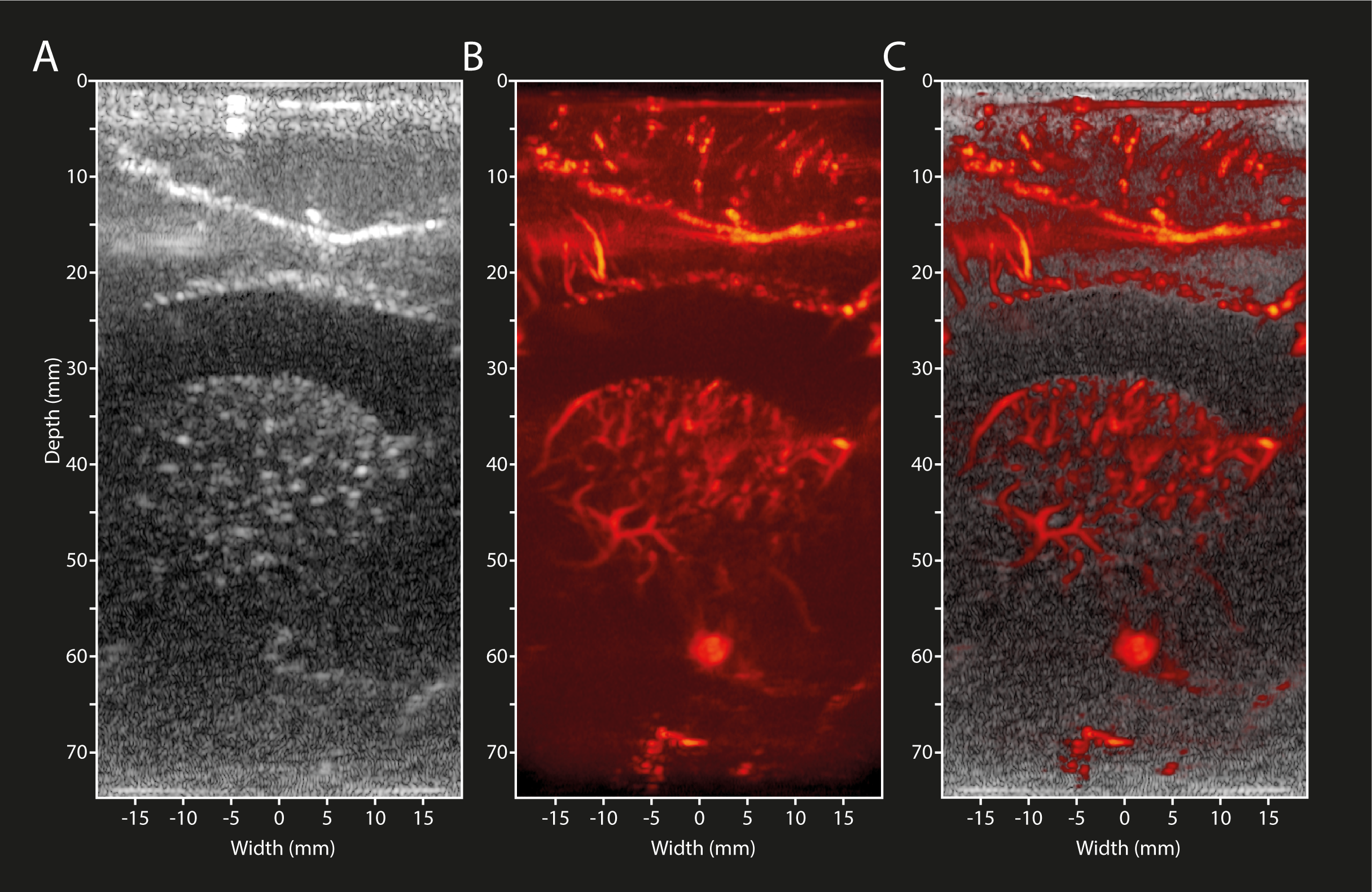

Supplement: FIGURE S3 — Vascular imaging of the thalamus in pt.#10. Incidental imaging session of the thalamus in pt.#10, who presented with a LGG in the left frontal lobe. (A) Conventional B-mode image in a sagittal plane, showing (from top to bottom) the corpus callosum, the ventricle, and the thalamus. (B) The PDI of the same field of view as described under (A), reveals a rich vascular pattern in the thalamus, allowing for a clear delineation of the nucleus from the surrounding brain tissue. This would open up possibilities for monitoring of vascularization of critical structures such as the thalamus during neurosurgical procedures. (C) An overlay of B-mode and PDI, reveals the vascular pattern in relation to the tissue as would be displayed in conventional echography. LGG, low grade glioma; PDI, power doppler image. [file Image_3.TIF]
