# Supplementary material for: Functional Ultrasound (fUS) During Awake Brain Surgery: The Clinical Potential of Intra-Operative Functional and Vascular Brain Mapping
Source: Front Neurosci. 2020 Jan 9;13:1384. doi: 10.3389/fnins.2019.01384 (PMC6962116; doi:10.3389/fnins.2019.01384)
Supplement: TABLE S1 — Overview of functional tasks as used in the current study. [file Table_1.DOCX]

**Supplementary Table 1 –** Overview of functional tasks as used in the current study.

| Task Type | Task | Goal of Task | Used in Pts |
| --- | --- | --- | --- |
| Motor | Lip Pouting | Functional response motor cortex of the mouth | 1 |
|  | Finger Tapping | Functional response motor cortex of the hand | 1, 3, 4, 9 |
| Visual | Checkerboard stimulus | Functional Response of visual cortex (occipital) | 6, 7 |
| Language | Sentence Repetition | Functional response language related areas | 2 |
|  | Word Repetition (Verbal) | Functional response language related areas | 5, 7, 8, 10 |
|  | Word Repetition (Non-verbal) | Functional response language related areas, control for involvement primary motor cortex of the mouth | 5, 6, 8, 10 |
