# Supplementary material for: Functional Ultrasound (fUS) During Awake Brain Surgery: The Clinical Potential of Intra-Operative Functional and Vascular Brain Mapping
Source: Front Neurosci. 2020 Jan 9;13:1384. doi: 10.3389/fnins.2019.01384 (PMC6962116; doi:10.3389/fnins.2019.01384)
Supplement: TABLE S2 — Overview of all recordings (n = 90) as performed in the context of the current study. [file Table_2.DOCX]

**Supplementary Table 2 –** Overview of all recordings (n=90) as performed in the context of the current study

| **ID** | **Patient** | **Content** | **Imaging Direction** | **Imaging Depth (in cm)** | **Number of Angles** | **Ensemble Size** | **PRF (Hz)** | **Raw FR (Hz)** | **Live Doppler FR (Hz)** | **Functional Task** | **ESM*** | **Mean absolute displacement in x-axis (in mm)** | **Functional Response?** | **PCC ‘r’ (min-max)** | **Comments** |
| --- | --- | --- | --- | --- | --- | --- | --- | --- | --- | --- | --- | --- | --- | --- | --- |
| 1 | 1 | Functional | Still | 30 | 16 | 120 | 8000 | 500 | 4,2 | Motor (Lip Pouting) | 3,4 | 0.010 | No |  |  |
| 2 | 1 | Functional | Still | 30 | 16 | 120 | 8000 | 500 | 4,2 | Motor (Lip Pouting) |  | 0.012 | No |  |  |
| 3 | 1 | Functional | Still | 30 | 16 | 120 | 8000 | 500 | 4,2 | Motor (Lip Pouting) |  | 0.008 | No |  |  |
| 4 | 1 | Functional | Still | 30 | 16 | 120 | 8000 | 500 | 4,2 | Motor (Finger Tapping) |  | 0.008 | No |  |  |
| 5 | 1 | Functional | Still | 30 | 16 | 120 | 8000 | 500 | 4,2 | Motor (Finger Tapping) |  | 0.145 | No |  |  |
| 6 | 1 | Vasculature; pre-resection | Linear sweep | 30 | 16 | 120 | 8000 | 500 | 4,2 |  |  |  |  |  |  |
| 7 | 1 | Functional | Still | 30 | 16 | 120 | 8000 | 500 | 4,2 | Motor (Lip Pouting) |  | 0.400 | No |  |  |
| 8 | 1 | Functional | Still | 30 | 16 | 120 | 8000 | 500 | 4,2 | Motor (Lip Pouting) |  | 0.286 | No |  |  |
| 9 | 1 | Functional | Still | 30 | 16 | 120 | 8000 | 500 | 4,2 | Motor (Lip Pouting) |  | 0.394 | Yes | 0.3-0.73 |  |
| 10 | 2 | Vasculature; pre-resection | Linear sweep | 50 | 12 | 120 | 6000 | 500 | 4,2 |  |  |  |  |  |  |
| 11 | 2 | Vasculature; pre-resection | Linear sweep | 50 | 12 | 120 | 6000 | 500 | 4,2 |  |  |  |  |  |  |
| 12 | 2 | Functional | Still | 50 | 12 | 120 | 6000 | 500 | 4,2 | Language (Sentence repetition) | 1,2,3 | 0.124 | No |  |  |
| 13 | 2 | Functional | Still | 50 | 12 | 120 | 6000 | 500 | 4,2 | Language (Sentence repetition) |  | 0.101 | Yes | 0.3-0.75 |  |
| 14 | 2 | Functional | Still | 50 | 12 | 120 | 6000 | 500 | 4,2 | Language (Sentence repetition) |  | 0.650 | No |  |  |
| 15 | 2 | Vasculature; post-resection | Linear sweep | 50 | 12 | 120 | 6000 | 500 | 4,2 |  |  |  |  |  |  |
| 16 | 2 | Vasculature; post-resection | Linear sweep | 50 | 12 | 120 | 6000 | 500 | 4,2 |  |  |  |  |  |  |
| 17 | 3 | Vasculature; pre-resection | Linear sweep | 50 | 12 | 120 | 6000 | 500 | 4,2 |  |  |  |  |  |  |
| 18 | 3 | Vasculature; pre-resection | Linear sweep | 50 | 12 | 120 | 6000 | 500 | 4,2 |  |  |  |  |  |  |
| 19 | 3 | Vasculature; pre-resection | Linear sweep | 50 | 12 | 120 | 6000 | 500 | 4,2 |  |  |  |  |  |  |
| 20 | 3 | Vasculature; pre-resection | Linear sweep | 50 | 12 | 120 | 6000 | 500 | 4,2 |  |  |  |  |  |  |
| 21 | 3 | Functional | Still | 50 | 12 | 120 | 6000 | 500 | 4,2 | Motor (Finger Tapping) | 4 | 0.084 | No |  | Measured pre-ESM |
| 22 | 3 | Functional | Still | 50 | 12 | 120 | 6000 | 500 | 4,2 | Motor (Finger Tapping) |  | 0.070 | No |  | Measured pre-ESM |
| 23 | 3 | Vasculature; post-resection | Linear sweep | 50 | 12 | 120 | 6000 | 500 | 4,2 |  |  |  |  |  |  |
| 24 | 4 | Vasculature; pre-resection | Linear sweep | 50 | 12 | 140 | 8000 | 667 | 4,8 |  |  |  |  |  |  |
| 25 | 4 | Vasculature; pre-resection | Fanning | 50 | 12 | 140 | 8000 | 667 | 4,8 |  |  |  |  |  |  |
| 26 | 4 | Functional | Still | 50 | 12 | 140 | 8000 | 667 | 4,8 | Motor (Finger Tapping) | 5 | 0.081 | Yes | 0.3-0.62 | Measured pre-ESM |
| 27 | 4 | Functional | Still | 50 | 12 | 140 | 8000 | 667 | 4,8 | Motor (Finger Tapping) |  | 0.024 | No |  | Measured pre-ESM |
| 28 | 4 | Functional | Still | 50 | 12 | 140 | 8000 | 667 | 4,8 | Motor (Finger Tapping) |  | 0.457 | No |  |  |
| 29 | 4 | Functional | Still | 50 | 12 | 140 | 8000 | 667 | 4,8 | Motor (Finger Tapping) |  | 0.347 | No |  |  |
| 30 | 4 | Vasculature; post-resection | Linear sweep | 50 | 12 | 140 | 8000 | 667 | 4,8 |  |  |  |  |  |  |
| 31 | 4 | Vasculature; post-resection | Fanning | 50 | 12 | 140 | 8000 | 667 | 4,8 |  |  |  |  |  |  |
| 32 | 5 | Vasculature; pre-resection | Linear sweep | 50 | 12 | 140 | 8000 | 667 | 4,8 |  |  |  |  |  |  |
| 33 | 5 | Vasculature; pre-resection | Fanning | 50 | 12 | 140 | 8000 | 667 | 4,8 |  |  |  |  |  |  |
| 34 | 5 | Vasculature; pre-resection | Fanning | 50 | 12 | 140 | 8000 | 667 | 4,8 |  |  |  |  |  |  |
| 35 | 5 | Vasculature; pre-resection | Fanning | 50 | 12 | 140 | 8000 | 667 | 4,8 |  |  |  |  |  |  |
| 36 | 5 | Functional | Still | 50 | 12 | 140 | 8000 | 667 | 4,8 | Language (Word Repetition-Verbal) | 1,6 | 0.078 | Yes | 0.3-0.72 |  |
| 37 | 5 | Functional | Still | 50 | 12 | 140 | 8000 | 667 | 4,8 | Language (Word Repetition-Silent) |  | 0.018 | Yes | 0.3-0.72 |  |
| 38 | 5 | Functional | Still | 50 | 12 | 140 | 8000 | 667 | 4,8 | Language (Word Repetition-Verbal) |  | 0.175 | No |  |  |
| 39 | 5 | Vasculature; post-resection | Linear sweep | 50 | 12 | 140 | 8000 | 667 | 4,8 |  |  |  |  |  |  |
| 40 | 5 | Vasculature; post-resection | Fanning | 50 | 12 | 140 | 8000 | 667 | 4,8 |  |  |  |  |  |  |
| 41 | 6 | Vasculature; pre-resection | Linear sweep | 50 | 12 | 140 | 8000 | 667 | 4,8 |  |  |  |  |  |  |
| 42 | 6 | Vasculature; pre-resection | Fanning | 50 | 12 | 140 | 8000 | 667 | 4,8 |  |  |  |  |  |  |
| 43 | 6 | Vasculature; pre-resection | Fanning | 50 | 12 | 140 | 8000 | 667 | 4,8 |  |  |  |  |  |  |
| 44 | 6 | Functional | Still | 50 | 12 | 140 | 8000 | 667 | 4,8 | Visual (Checkerboard) | No functional deficits in ESM | 0.058 | No |  |  |
| 45 | 6 | Functional | Still | 50 | 12 | 140 | 8000 | 667 | 4,8 | Visual (Checkerboard) |  | 0.641 | No |  |  |
| 46 | 6 | Functional | Still | 50 | 12 | 140 | 8000 | 667 | 4,8 | Visual (Checkerboard) |  | 0.189 | No |  |  |
| 47 | 6 | Functional | Still | 50 | 12 | 140 | 8000 | 667 | 4,8 | Language (Word Repetition-Covert) |  | 0.480 | No |  |  |
| 48 | 6 | Functional | Still | 50 | 12 | 140 | 8000 | 667 | 4,8 | Language (Word Repetition-Covert) |  | 0.075 | No |  |  |
| 49 | 6 | Vasculature; post-resection | Linear sweep | 50 | 12 | 140 | 8000 | 667 | 4,8 |  |  |  |  |  |  |
| 50 | 6 | Vasculature; post-resection | Linear sweep | 50 | 12 | 140 | 8000 | 667 | 4,8 |  |  |  |  |  |  |
| 51 | 7 | Vasculature; pre-resection | Linear sweep | 50 | 12 | 140 | 8000 | 667 | 4,8 |  |  |  |  |  |  |
| 52 | 7 | Vasculature; pre-resection | Linear sweep | 50 | 12 | 140 | 8000 | 667 | 4,8 |  |  |  |  |  |  |
| 53 | 7 | Vasculature; pre-resection | Linear sweep | 50 | 12 | 140 | 8000 | 667 | 4,8 |  |  |  |  |  |  |
| 54 | 7 | Vasculature; pre-resection | Fanning | 50 | 12 | 140 | 8000 | 667 | 4,8 |  |  |  |  |  |  |
| 55 | 7 | Vasculature; pre-resection | Fanning | 50 | 12 | 140 | 8000 | 667 | 4,8 |  |  |  |  |  |  |
| 56 | 7 | Functional | Still | 50 | 12 | 140 | 8000 | 667 | 4,8 | Visual (Checkerboard) | No functional deficits in ESM | 0.199 | No |  |  |
| 57 | 7 | Functional | Still | 50 | 12 | 140 | 8000 | 667 | 4,8 | Visual (Checkerboard) |  | 0.118 | No |  |  |
| 58 | 7 | Functional | Still | 50 | 12 | 140 | 8000 | 667 | 4,8 | Visual (Checkerboard) |  | 0.382 | No |  |  |
| 59 | 7 | Functional | Still | 50 | 12 | 140 | 8000 | 667 | 4,8 | Language (Word Repetition-Verbal) |  | 0.208 | Yes | 0.3-0.62 |  |
| 60 | 7 | Functional | Still | 50 | 12 | 140 | 8000 | 667 | 4,8 | Language (Word Repetition-Verbal) |  | 0.216 | No |  |  |
| 61 | 7 | Vasculature; post-resection | Linear sweep | 50 | 12 | 140 | 8000 | 667 | 4,8 |  |  |  |  |  |  |
| 62 | 7 | Vasculature; post-resection | Linear sweep | 50 | 12 | 140 | 8000 | 667 | 4,8 |  |  |  |  |  |  |
| 63 | 8 | Vasculature; pre-resection | Fanning | 50 | 12 | 140 | 8000 | 667 | 4,8 |  |  |  |  |  |  |
| 64 | 8 | Vasculature; pre-resection | Fanning | 50 | 12 | 140 | 8000 | 667 | 4,8 |  |  |  |  |  |  |
| 65 | 8 | Vasculature; pre-resection | Fanning | 80 | 12 | 140 | 6000 | 500 | 3,6 |  |  |  |  |  |  |
| 66 | 8 | Functional | Still | 50 | 12 | 140 | 8000 | 667 | 4,8 | Language (Word Repetition-Verbal) | No functional deficits in ESM | 0.255 | No |  |  |
| 67 | 8 | Functional | Still | 50 | 12 | 140 | 8000 | 667 | 4,8 | Language (Word Repetition-Verbal) |  | 0.861 | No |  |  |
| 68 | 8 | Functional | Still | 50 | 12 | 140 | 8000 | 667 | 4,8 | Language (Word Repetition-Covert) |  | 0.111 | No |  |  |
| 69 | 8 | Functional | Still | 50 | 12 | 140 | 8000 | 667 | 4,8 | Language (Word Repetition-Covert) |  | 0.573 | No |  |  |
| 70 | 8 | Functional | Still | 75 | 12 | 140 | 6000 | 500 | 3,6 | Motor (Finger Tapping) |  | 0.124 | No |  | Post-resection |
| 71 | 8 | Vasculature; post-resection | Fanning | 75 | 12 | 140 | 6000 | 500 | 3,6 |  |  |  |  |  |  |
| 72 | 9 | Vasculature; pre-resection | Linear sweep | 50 | 12 | 140 | 8000 | 667 | 4,8 |  | 4,5,7 |  |  |  |  |
| 73 | 9 | Vasculature; pre-resection | Linear sweep | 50 | 12 | 140 | 8000 | 667 | 4,8 |  |  |  |  |  |  |
| 74 | 9 | Vasculature; pre-resection | Linear sweep | 75 | 12 | 140 | 6000 | 500 | 3,6 |  |  |  |  |  |  |
| 75 | 9 | Functional | Still | 50 | 12 | 140 | 8000 | 667 | 4,8 | Motor (Finger Tapping) |  | 0.093 | No |  |  |
| 76 | 9 | Functional | Still | 50 | 12 | 140 | 8000 | 667 | 4,8 | Motor (Finger Tapping) |  | 0.210 | No |  |  |
| 77 | 9 | Functional | Still | 50 | 12 | 140 | 8000 | 667 | 4,8 | Motor (Finger Tapping) |  | 0.032 | Yes | 0.3-0.72 | Contralateral hand |
| 78 | 9 | Functional | Still | 75 | 12 | 140 | 6000 | 500 | 3,6 | Motor (Finger Tapping) |  | 0.608 | No |  |  |
| 79 | 9 | Functional | Still | 75 | 12 | 140 | 6000 | 500 | 3,6 | Motor (Finger Tapping) |  | 0.206 | No |  |  |
| 80 | 9 | Vasculature; post-resection | Linear sweep | 75 | 12 | 140 | 6000 | 500 | 3,6 |  |  |  |  |  |  |
| 81 | 9 | Vasculature; post-resection | Fanning | 75 | 12 | 140 | 6000 | 500 | 3,6 |  |  |  |  |  |  |
| 82 | 10 | Vasculature; pre-resection | Linear sweep | 50 | 12 | 140 | 8000 | 667 | 4,8 |  |  |  |  |  |  |
| 83 | 10 | Vasculature; pre-resection | Fanning | 50 | 12 | 140 | 8000 | 667 | 4,8 |  |  |  |  |  |  |
| 84 | 10 | Vasculature; pre-resection | Fanning | 50 | 12 | 140 | 8000 | 667 | 4,8 |  |  |  |  |  |  |
| 85 | 10 | Functional | Still | 50 | 12 | 140 | 8000 | 667 | 4,8 | Language (Word Repetition-Verbal) | No functional deficits in ESM | 0.263 | No |  |  |
| 86 | 10 | Functional | Still | 50 | 12 | 140 | 8000 | 667 | 4,8 | Language (Word Repetition-Covert) |  | 0.164 | No |  |  |
| 87 | 10 | Functional | Still | 50 | 12 | 140 | 8000 | 667 | 4,8 | Language (Word Repetition-Verbal) |  | 0.171 | Yes | 0.3-0.72 |  |
| 88 | 10 | Functional | Still | 50 | 12 | 140 | 8000 | 667 | 4,8 | Language (Word Repetition-Covert) |  | 0.020 | Yes | 0.3-0.72 |  |
| 89 | 10 | Vasculature; post-resection | Fanning | 75 | 12 | 140 | 6000 | 500 | 3,6 |  |  |  |  |  |  |
| 90 | 10 | Vasculature; post-resection | Linear sweep | 75 | 12 | 140 | 6000 | 500 | 3,6 |  |  |  |  |  |  |

** 1= phonemic paraphasia, 2= anomia (anomic aphasia), 3= motor cortex mouth, 4= motor cortex hand, 5= motor cortex arm, 6= aphasia (speech arrest), 7= motor cortex wrist*

*ESM= Electrocortical Stimulation Mapping, PRF=Pulse Repetition Frequency, FR= Frame Rate, PCC= Pearson Correlation Coefficient*
